# Discriminant COVID-19 Outcomes Based on Safety Adherence in an Active Lifestyle Retirement Community

**DOI:** 10.1093/geroni/igab046.2697

**Published:** 2021-12-17

**Authors:** Mitchell Roberts, Erica Sappington, Leonardo Guerra, Lindsey Collins, Ali Yalcin, Carla VandeWerd, Jeff Lowenkron

**Affiliations:** 1 University of South Florida, Tampa, Florida, United States; 2 University of South Florida, University of South Florida, Florida, United States; 3 University of Florida, University of Florida, Florida, United States; 4 The Villages Health, The Villages, Florida, United States

## Abstract

Compliance with preventive behaviors recommended by public health officials plays a critical role in the control and prevention of COVID-19. Data were collected from those living in The Villages, FL, and surrounding communities via The Villages Health COVID-19 Rapid Testing Program in partnership with The UFHealth Precision Health Research Center. A descriptive ecological study was conducted to model COVID-19 positivity result variations by age, sex and adherence to CDC safety recommendations using chi-square tests. 9,993 tests were performed using Abbott's BinaxNOW™ COVID-19 Ag Card, and 931 (9.30%) positive cases were confirmed between 10/19/2020-2/26/2021. Median age was 69 years (range:12-103), and 5,578 (55.8%) individuals were female. No significant differences were found in positive test status (≥65=9.8%,<65=8.8%) amongst those over 65 (n=6567) and under 65 (n=3180) years old [X2 (1, N=9847)=2.49,p=.114]; however, positive test result differed by sex with males (10.6%) testing positive at higher rates than females [8.3%, X2(1, N =9993)=14.888, p< .001)]. A significant relationship between preventative behaviors and positive test status was also found. Not engaging in regular handwashing (p< .001) and failing to stay 6 feet or more away from others outdoors (p< .001) was significantly associated with positive test status. Further, not wearing a face mask in businesses/shops (p<.001) or indoors around 6+ people, (p<.001) was significantly associated with positive test status. In light of debate around the efficacy of mask wearing, these findings signal the importance of following CDC recommended public health behaviors for all ages across the lifespan to reduce the spread of COVID-19 infection.

